# Study of energy transfer mechanism from ZnO nanocrystals to Eu^3+^ ions

**DOI:** 10.1186/s11671-016-1282-3

**Published:** 2016-02-09

**Authors:** Vivek Mangalam, Kantisara Pita, Christophe Couteau

**Affiliations:** OPTIMUS, Centre for OptoElectronics and Biophotonics, School of Electrical and Electronic Engineering, Nanyang Technological University (NTU), Block S2, 50 Nanyang Avenue, Singapore, 639798 Singapore; CINTRA, CNRS-NTU-Thales UMI 3288, Research Techno Plaza, 50 Nanyang Drive, Border X Block, Level 6, Singapore, 637553 Singapore; Laboratory for Nanotechnology, Instrumentation and Optics (LNIO), Charles Delaunay Institute CNRS UMR 6281, University of Technology of Troyes (UTT), 12 rue Marie Curie, 10000 Troyes, France; School of Electrical and Electronic Engineering, Nanyang Technological University (NTU), Block S2, 50 Nanyang Avenue, Singapore, 639798 Singapore

**Keywords:** Zinc oxide nanocrystals, Energy transfer mechanism, Europium(III) ions, Photoluminescence

## Abstract

In this work, we investigate the efficient energy transfer occurring between ZnO nanocrystals (ZnO-nc) and europium (Eu^3+^) ions embedded in a SiO_2_ matrix prepared using the sol-gel technique. We show that a strong red emission was observed at 614 nm when the ZnO-nc were excited using a continuous optical excitation at 325 nm. This emission is due to the radiative ^5^D_0_ → ^7^F_2_ de-excitation of the Eu^3+^ ions and has been conclusively shown to be due to the energy transfer from the excited ZnO-nc to the Eu^3+^ ions. The photoluminescence excitation spectra are also examined in this work to confirm the energy transfer from ZnO-nc to the Eu^3+^ ions. Furthermore, we study various de-excitation processes from the excited ZnO-nc and their contribution to the energy transfer to Eu^3+^ ions. We also report the optimum fabrication process for maximum red emission at 614 nm from the samples where we show a strong dependence on the annealing temperature and the Eu^3+^ concentration in the sample. The maximum red emission is observed with 12 mol% Eu^3+^ annealed at 450 °C. This work provides a better understanding of the energy transfer mechanism from ZnO-nc to Eu^3+^ ions and is important for applications in photonics, especially for light emitting devices.

## Background

In recent years, there has been a lot of interest in having a strong interaction between semiconductor nanocrystals and rare earth (RE) ions [1, 2]. Semiconductor nanocrystals have been used as sensitizers to excite RE ions due to their intrinsic properties such as size-dependent luminescent properties [3], large absorption cross sections [4–6] and broad excitation spectra [6]. In this process, semiconductor nanocrystals are excited using a light source and transfer the energy to the RE ions which can then reemit light at a different wavelength. Understanding the energy transfer mechanisms between semiconductor nanocrystals and RE ions helps in developing energy-efficient light sources such as white light sources [7], fibre amplifiers [4, 5], field emission displays [5, 8], fluorescent lamps and solid state lasers [5].

The energy transfer between several types of semiconductor nanocrystals and RE ions, such as silicon nanocrystals (Si-nc) and Er^3+^ ions [9], ZnO-nc and Ce^3+^ ions [5], ZnO-nc and Er^3+^ ions [10], and ZnO-nc and Tb^3+^ ions [11], have been studied. The energy transfer between ZnO-nc and Eu^3+^ ion has also been observed [6, 7, 12–16], and this system has been investigated because of the usefulness of the sharp red emission from Eu^3+^ ions centred at 614 nm. The Eu^3+^ ions are usually either co-doped with ZnO-nc in a dielectric host matrix like SiO_2_ [6, 16] or embedded inside the ZnO-nc [7, 12–15]. Co-doping of ZnO-nc and Eu^3+^ ions in a dielectric matrix is preferable due to the physical and chemical protection it provides to the dopants [17]. While some studies [6, 12, 13, 16] report that the energy transfer take place with the involvement of the defect states in ZnO-nc, others [14, 15] report that this energy transfer is predominantly due to the free or bound excitonic state emissions of ZnO-nc. However, a comprehensive understanding of this energy transfer mechanism and the contribution of energy transfer from the various ZnO-nc emissions has not been reported and thus needs to be investigated. In particular, this is important to develop efficient ZnO-nc and Eu^3+^ ion light-emitting devices and also make an important contribution to have a better understanding of the energy transfer processes from nanocrystals to RE ions, in general.

In this article, we present in detail the contribution of the various de-excitation processes of excited ZnO-nc embedded in SiO_2_ matrix in the energy transfer process from ZnO-nc to Eu^3+^ ions and we suggest a suitable mechanism for the energy transfer process. This is an extension of our earlier work [18], where the various de-excitation processes of the ZnO-nc in SiO_2_ were identified as being made of seven contributions. In this work, the low-cost sol-gel process was used to make the samples due to the flexibility of controlling the material composition and the structures of the thin film. Fabrication parameters of this technique like annealing temperature and Eu^3+^ ion concentration have been studied and optimised to achieve maximum red emission from the Eu^3+^ ions. We are then able to provide the best parameters in order to get the strongest energy transfer and thus get the strongest red emission at 614 nm.

## Methods

The low-cost sol-gel technique was used to prepare three different types of samples, namely Eu^3+^ ions incorporated in SiO_2_ matrix (Eu^3+^:SiO_2_); ZnO nanocrystals embedded in SiO_2_ matrix (ZnO-nc:SiO_2_) and Eu^3+^ ions and ZnO-nc incorporated in SiO_2_ matrix (Eu^3+^_*x*_:(ZnO-nc:SiO_2_) where *x* is the concentration of Eu^3+^ ions in molar fraction, and calculated using $x=\frac{\mathrm{moles}\ \mathrm{of}\ \left({\mathrm{Eu}}^{3+}\right)}{\mathrm{moles}\ \mathrm{of}\ \left({\mathrm{Eu}}^{3+}+\mathrm{Z}\mathrm{n}+\mathrm{S}\mathrm{i}\right)}$). Different Eu^3+^_*x*_:(ZnO-nc:SiO_2_) samples were prepared with *x* ranging from 0.04 to 0.16. For ZnO-nc:SiO_2_ and Eu^3+^_*x*_:(ZnO-nc:SiO_2_) samples, the Zn:Si molar ratio was maintained at 1:2. For the Eu^3+^:SiO_2_ sample, the molar ratio of Eu^3+^:Si was kept the same as that in the Eu^3+^_0.12_:(ZnO-nc:SiO_2_) sample. The preparation method used for the above samples is similar to that described in our previous publication [18]. In the first step of the three-step process, the precursor, the solvent and the catalyst were mixed to create the sol. Two different sols for SiO_2_ matrix and ZnO-nc were developed from tetraethyl orthosilicate (TEOS) and zinc acetate as precursors, respectively. To incorporate the Eu^3+^ ions, europium(III) nitrate was added into the TEOS sol after ageing the sol for 24 h. The two sols were then mixed together and spin coated on a (100) Si wafer substrate. These samples were soft baked and were then annealed using rapid thermal processing (RTP) at various annealing temperatures ranging from 450 to 600 °C for 1 min in an O_2_ environment. The formation of ZnO-nc in SiO_2_ using this fabrication recipe was previously studied and verified using TEM images [18]. These samples had a thickness of approximately 300 nm, which was measured using a Dektak 3 profilometer. The characterisation of the samples was done by studying room-temperature photoluminescence (PL) emission spectra and photoluminescence excitation (PLE) spectra using a spectrofluorometer (SPEX Fluorolog-3 Model FL3-11). For the PL emission spectra, the samples were excited at 325 nm using a 450-W xenon short arc lamp coupled to a monochromator and a 325 nm line filter, while the PLE spectra were obtained by measuring the 614 nm emission intensity of the samples while varying the excitation wavelength from 325 to 550 nm using the monochromator.

## Results and discussion

**Fig. 1:**
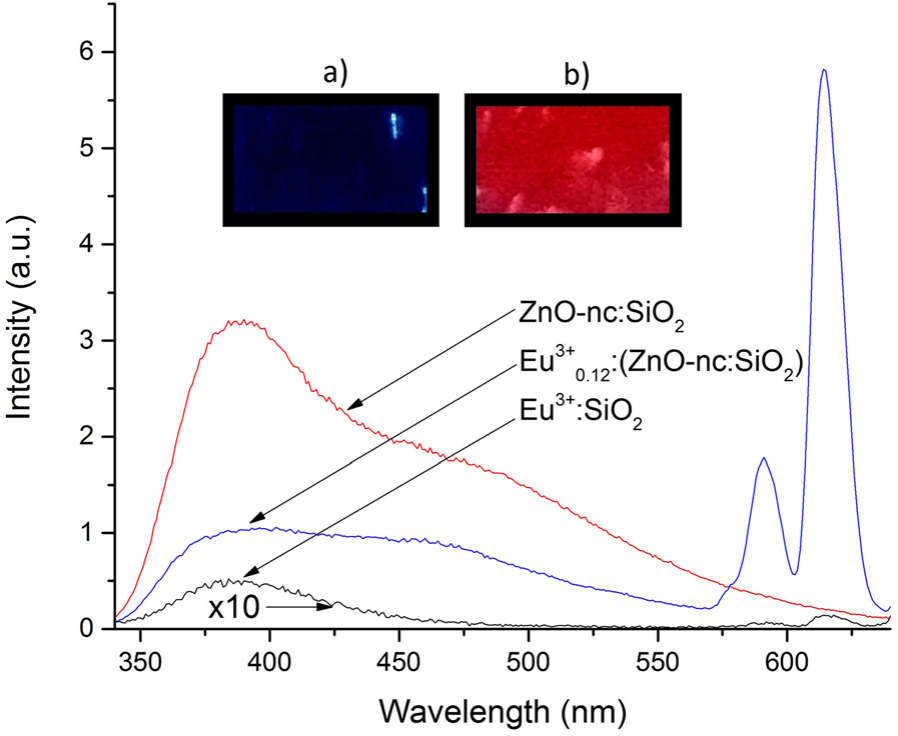
PL spectra of Eu^3+^:SiO_2_, ZnO-nc:SiO_2_ and Eu^3+^_0.12_:(ZnO-nc:SiO_2_) samples. *Insets a)* and *b)* show, respectively, the photograph of Eu^3+^:SiO_2_ and Eu^3+^_0.12_:(ZnO-nc:SiO_2_) samples excited using a mercury vapour UV lamp

**Fig. 2:**
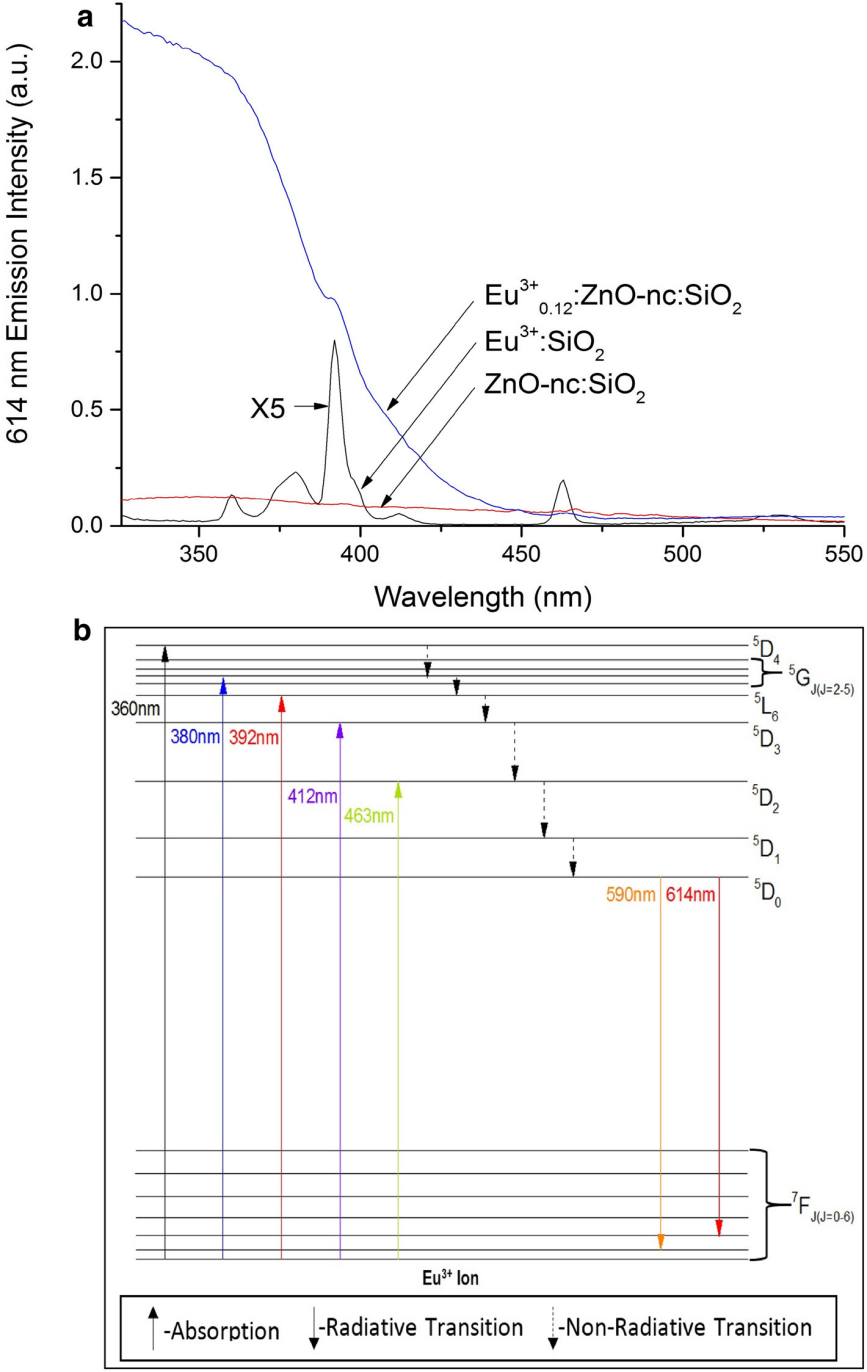
**a** PLE spectra of Eu^3+^:SiO_2_, ZnO-nc:SiO_2_ and Eu^3+^_0.12_:(ZnO-nc:SiO_2_) samples. **b** Schematic Dieke energy level diagram of the Eu^3+^ ions in SiO_2_

The PL spectra of Eu^3+^:SiO_2_, ZnO-nc:SiO_2_ and Eu^3+^_0.12_:(ZnO-nc:SiO_2_) samples annealed at 450 °C using RTP are shown in Fig. 1. The PL emission of the SiO_2_ film alone (not shown) prepared using the sol-gel method showed negligible emission, which indicates that the spectra of the samples shown in Fig. 1 are not affected by the presence of the host SiO_2_ matrix. Firstly, we observe that the ZnO-nc:SiO_2_ sample shows a broadband emission from the ZnO-nc. The ZnO-nc broadband emission follows a trend similar to the one in our previous study [18]. We also observe that the Eu^3+^:SiO_2_ sample shows negligible emission at all wavelengths including at 614 nm. This is a strong evidence that the optical excitation at 325 nm does not directly excite the Eu^3+^ ions in the sample [19]. The Eu^3+^_0.12_:(ZnO-nc:SiO_2_) sample, however, shows a broadband emission along with two high-intensity sharp peaks at 590 and 614 nm. The 590 and 614 nm emissions are known as the ^5^D_0_ → ^7^F_1_ and ^5^D_0_ → ^7^F_2_ transitions from the Eu^3+^ ions [19] (see the energy level diagram in Fig. 2b). These results clearly demonstrate that the 325 nm light source excites the ZnO-nc which can then transfer the energy to the Eu^3+^ ions which in turn gives a strong emission in the red. In addition, we can see that the intensity of the broadband emission from the Eu^3+^_0.12_:(ZnO-nc:SiO_2_) sample between 350 and 575 nm is lower than that from the ZnO-nc:SiO_2_ sample, indicating that the reduction of the emission intensity is due to the energy transfer from ZnO-nc to the Eu^3+^ ions. The insets (a and b) in Fig. 1 show, respectively, the photograph of Eu^3+^:SiO_2_ and Eu^3+^_0.12_:(ZnO-nc:SiO_2_) samples optically excited using a mercury vapour UV lamp. The bright red emission, due to the ZnO-nc mediated excitation of the Eu^3+^ ions, is clearly visible from the Eu^3+^_0.12_:(ZnO-nc:SiO_2_) sample.

Further evidence of energy transfer from ZnO-nc to the Eu^3+^ ions is seen from the PLE spectra of Eu^3+^:SiO_2_, ZnO-nc:SiO_2_ and Eu^3+^_0.12_:(ZnO-nc:SiO_2_) samples annealed at 450 °C using RTP shown in Fig. 2a. As mentioned above in the ‘Methods’ section, the PLE spectra gives the measure of the 614 nm red emission intensity of the samples as function of varying excitation wavelengths. Firstly, we note that the PLE spectrum of the ZnO-nc:SiO_2_ shows a very low 614 nm emission at all excitation wavelengths. This very low 614 nm emission of the ZnO-nc:SiO_2_ sample is due to the slight oxygen defect emission from ZnO-nc (explained in Fig. 4), upon excitation of ZnO-nc. Figure 2a also shows the PLE spectrum of Eu^3+^:SiO_2_ sample, in which we observe the five characteristic excitation peaks of the Eu^3+^ ions centred at 360, 380, 392, 412 and 463 nm which are due to ^7^F_0_ → ^5^D_4_, ^7^F_0_ → ^5^G_*J*(*J* = 2−5)_, ^7^F_0_ → ^5^L_6_, ^7^F_0_ → ^5^D_3_ and ^7^F_0_ → ^5^D_2_ transitions [19] of Eu^3+^ ions, respectively. The various excitation peaks of the Eu^3+^ ions in SiO_2_ are represented schematically in the Dieke energy level diagram [20] in Fig. 2b. The Eu^3+^ ions in this sample upon excitation at the five peak wavelengths directly get excited and subsequently relax to the ground state through the radiative emission at 614 nm. Interestingly, the PLE spectra of the Eu^3+^_0.12_:(ZnO-nc:SiO_2_) sample shows a strong and broad 614 nm emission profile for excitation wavelengths between 325 and 370 nm, which then reduces till 450 nm. In this broad range, the 614 nm emission of the Eu^3+^_0.12_:(ZnO-nc:SiO_2_) sample is much greater than that of both Eu^3+^:SiO_2_ and ZnO-nc:SiO_2_ samples. This is due to the fact that upon excitation at wavelength less than 450 nm, the ZnO-nc in the Eu^3+^_0.12_:(ZnO-nc:SiO_2_) sample were excited which then transferred the energy to the Eu^3+^ ions in the sample through the various excitation peaks of the Eu^3+^ ions at 360, 380, 392, 412 and 463 nm. The excited Eu^3+^ ions subsequently relaxed to the ground states giving the red emission at 614 nm. We can also observe two other striking features for the PLE of Eu^3+^_0.12_:(ZnO-nc:SiO_2_) sample. First of all, there is a bit of a plateau between 325 and 375 nm for the PLE and this is because in this range, we are exciting the ZnO-nc above its band gap where all of the ZnO-nc emission centres [18] (explained in Fig. 4) are excited, thus resulting in large energy transfer to the Eu^3+^ ions. Between 375 and 450 nm, as we go below the band gap of ZnO, lesser and lesser ZnO-nc emission centres are excited which results in lesser energy transfer to the Eu^3+^ ion. The second feature is that we see a slight bump at 392 nm for the PLE of Eu^3+^_0.12_:(ZnO-nc:SiO_2_) due to the direct strong absorption line of the Eu^3+^ ions noticeable in the PLE of Eu^3+^:SiO_2_, this is in addition to the contribution of ZnO emission centres at this wavelength. Thus, these results from the PLE study undoubtedly confirms the energy transfer from ZnO-nc to the Eu^3+^ ions which was also observed from the PL spectra of the samples.

**Fig. 3:**
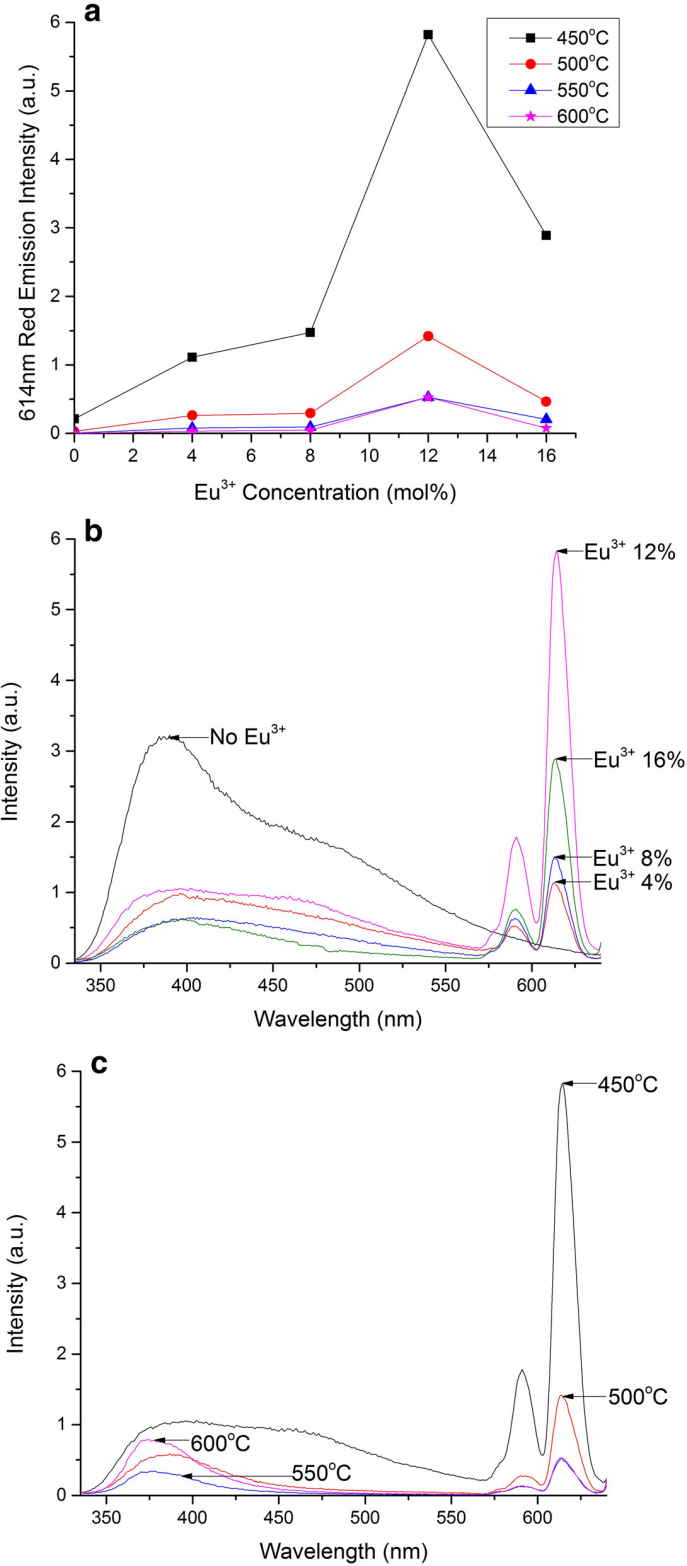
PL spectra and 614 nm red emission intensity from the Eu^3+^_*x*_:(ZnO-nc:SiO_2_) samples. **a** 614 nm red emission intensity from the Eu^3+^_*x*_:(ZnO-nc:SiO_2_) samples as a function of Eu^3+^ ion concentration at various annealing temperatures. **b** The PL spectra of the Eu^3+^_*x*_:(ZnO-nc:SiO_2_) samples RTP annealed at 450 °C for various Eu^3+^ ion concentrations. **c** The PL spectra of the Eu^3+^_0.12_:(ZnO-nc:SiO_2_) samples annealed by RTP at various temperatures

To obtain the optimum red emission intensity from the Eu^3+^_*x*_:(ZnO-nc:SiO_2_) configuration, different samples with various annealing temperatures and different Eu^3+^ ion concentrations were fabricated and studied. Figure 3a presents the intensity of emission at 614 nm from the Eu^3+^_*x*_:(ZnO-nc:SiO_2_) samples as a function of Eu^3+^ ion concentration (*x* ranging from 0 to 0.16) at various annealing temperatures from 450 to 600 °C. The corresponding PL spectra of the Eu^3+^_*x*_:(ZnO-nc:SiO_2_) samples, with *x* ranging from 0 to 0.16, annealed at 450 °C is shown in Fig. 3b. Here, we observe that the red emission intensity shows a non-linear increase with increasing the Eu^3+^ ion concentration from 0 to 12 mol%. This is expected as increasing the Eu^3+^ ions decreases the distance between the ZnO-nc and the Eu^3+^ ions which results in enhanced energy transfer [6, 16]. Thus, greater fraction of Eu^3+^ ions are excited with increasing concentration resulting in enhanced red emission. A further increase in Eu^3+^ ion concentration to 16 mol% shows a decrease in the 614 nm emission intensity. This is attributed to Eu^3+^ ion concentration quenching [21], i.e. migration of energy amongst the Eu^3+^ ions which is non-radiatively dissipated through the quenching sites. The close proximity of the Eu^3+^ ions due to increasing concentration results in concentration quenching. This trend is observed in all the Eu^3+^_*x*_:(ZnO-nc:SiO_2_) samples which were annealed at 450, 500, 550 and 600 °C. It is clearly shown here that the optimum Eu^3+^ ion concentration for maximum red emission is 12 mol%. This was also observed in Y_2_O_3_:Eu^3+^ thin film phosphors [22]. In Fig. 3a, we note that there is a small emission at 614 nm from the 0 mol% Eu^3+^ sample (i.e. ZnO-nc:SiO_2_ sample) annealed at 450 °C; this is due to the broadband nature of the ZnO-nc emission at this annealing temperature.

In Fig. 3c, the corresponding PL spectra of the Eu^3+^_0.12_:(ZnO-nc:SiO_2_) samples RTP annealed at temperatures ranging from 450 to 600 °C is also shown. Here, we observe that increasing the annealing temperature leads to a reduction in the red emission intensity from the samples. This is due to the change in the nature of the emissions from the ZnO-nc embedded in the samples with increasing annealing temperature. Energy is transferred from the ZnO-nc to the Eu^3+^ ions due to the overlap of the broadband ZnO-nc emission spectra [18] and Eu^3+^ ion excitation spectra (see Fig. 2a). Within the emission range of ZnO-nc from 350 to 575 nm, we see that there is a strong overlap with Eu^3+^ ion absorption which is responsible for the resonant energy transfer from the ZnO-nc to the Eu^3+^ ions in the samples. The energy transfer from ZnO-nc results in the excitation of the Eu^3+^ ions from their ground state (^7^F_0_) to any of the higher states (^5^D_2_, ^5^D_3_, ^5^D_4_, ^5^G_*J*(*J* = 2−5)_, ^5^L_6_) which subsequently relax back to their ground state by the radiative emissions in the red at 614 and 590 nm (see the energy level diagram in Fig. 2b). Since the broadband emission from the ZnO-nc is the largest at 450 °C annealing, the energy transfer will also be the strongest at this annealing temperature. By the same token, when the annealing temperature increases, the bandwidth of the broad emission from ZnO-nc decreases, thus resulting in decreasing the spectral overlap between the ZnO-nc broadband emission and the Eu^3+^ ion excitation and therefore a reduction in energy transfer from the ZnO-nc to the Eu^3+^ ions leading to a reduction in the red emission intensity from the Eu^3+^ ions. In addition, the energy transfer from ZnO-nc to the Eu^3+^ ions is inhibited in the samples annealed at 550 and 600 °C due to the possible formation of Zn_2_SiO_4_ at the surface of the ZnO-nc [23]. Formation of Zn_2_SiO_4_ reduces the size of ZnO-nc causing reduction of PL emission [23] from the ZnO-nc and also results in an increase in distance between ZnO-nc and Eu^3+^ ion which results in a reduction of energy transfer from the ZnO-nc to the Eu^3+^ ions. For our process, the optimum RTP annealing temperature has been found to be 450 °C.

**Fig. 4:**
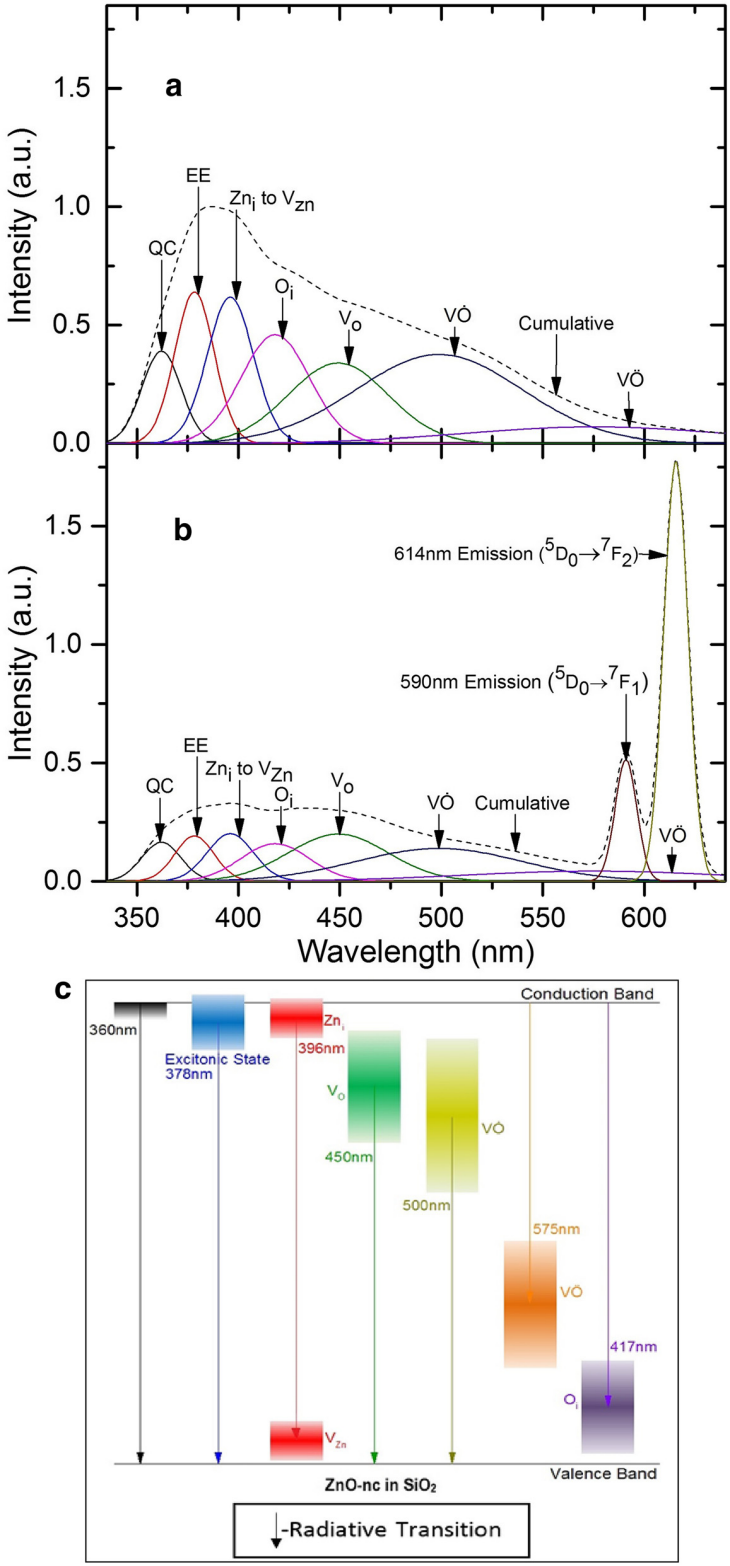
The emission centres of ZnO-nc in *(a)* ZnO-nc:SiO_2_ and *(b)* Eu^3+^_0.12_:(ZnO-nc:SiO_2_) samples annealed at 450 °C. See the main text for the various ZnO-nc emission centres. *(c)* The schematic energy level diagram of the emission centres of ZnO-nc in SiO_2_

In our previous work [18], we showed that the broad emission spectra of ZnO-nc embedded in SiO_2_ consists of seven Gaussian peaks centred at 360, 378, 396, 417, 450, 500 and 575 nm. The origins of these emissions have been discussed in reference [18]. The 360 and 378 nm peaks were attributed, respectively, to band edge emission from the smallest ZnO-nc which possibly experiences quantum confinement effect (labelled as QC) [24] and ZnO-nc excitonic emission (labelled as EE) [25, 26]. The 396 nm peak was attributed to the defect state electronic transition from Zn interstitial (labelled as Zn_i_) to Zn vacancy (labelled as V_Zn_) [17] and the remaining emission peaks were due to the electronic transition from, or to, the oxygen-related defects, namely oxygen interstitial (labelled as O_i_) defect emission at 417 nm [27, 28], oxygen vacancy (labelled as V_O_) emission at 450 nm, singly ionised oxygen vacancy (labelled as VȮ) emission at 500 nm and doubly charged oxygen vacancy (labelled as VÖ) emission at 575 nm [17, 29, 30]. The schematic energy level diagram of the seven ZnO-nc emission centres is shown in Fig. 4c. The energies of ZnO-nc emission centres are shown as broad vertical energy bands shown with a colour gradient to indicate the broad emission bandwidth of the various ZnO-nc emission centres. In this paper, we analyse the contribution of each of these emissions or de-excitation centres in exciting the Eu^3+^ ions. In order to do so, the PL emission spectra of the ZnO-nc:SiO_2_ and Eu^3+^_0.12_:(ZnO-nc:SiO_2_) samples annealed at 450 °C were deconvoluted using the seven de-excitation centres of ZnO-nc as shown in Fig. 4a, b, respectively. The fitting parameters, such as peak wavelength and full width at half maxima of the Gaussian peaks, were kept the same as those in our previous publication [18].

**Fig. 5:**
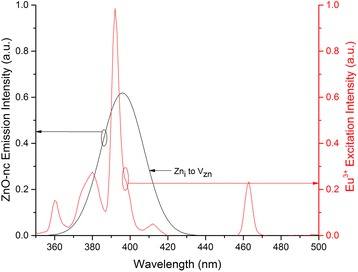
An example of the spectral overlap. The spectral overlap of Zn_i_ to V_Zn_ defect state emission of ZnO-nc:SiO_2_ sample annealed at 450 °C along with the Eu^3+^ excitation spectrum

To study the contribution of energy transfer from the ZnO-nc emission centres to the Eu^3+^ ions the spectral overlap integral value of emission from each of the seven ZnO-nc emission centres in ZnO-nc:SiO_2_ sample with the Eu^3+^ excitation spectrum was firstly determined. The spectral overlap integral value gives a measure of the radiative energy transfer from the ZnO-nc emission centres to the Eu^3+^ ions. An example of the spectral overlap between Zn_i_ and V_zn_ defect state emission of ZnO-nc:SiO_2_ sample annealed at 450 °C and the Eu^3+^ excitation are shown in Fig. 5. The spectral overlap integral from each of the seven ZnO-nc emission centres is shown in Fig. 7 on the left axis (solid line).

**Fig. 6:**
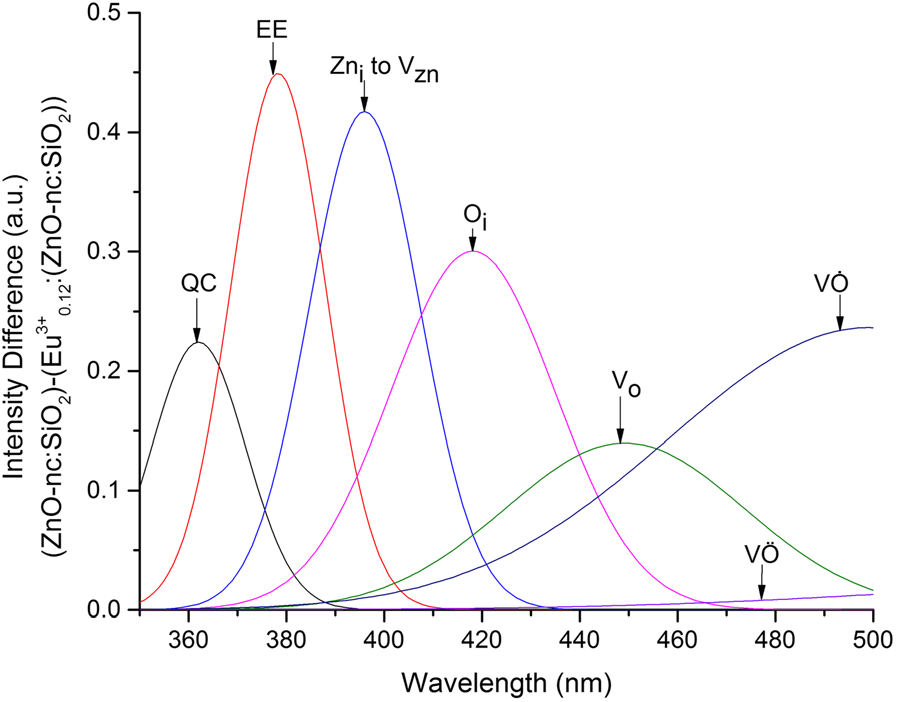
The intensity difference from ZnO-nc emission centres. The intensity difference between ZnO-nc:SiO_2_ and Eu^3+^_0.12_:(ZnO-nc:SiO_2_) samples annealed at 450 °C which is due to the incorporation of Eu^3+^ ions

**Fig. 7:**
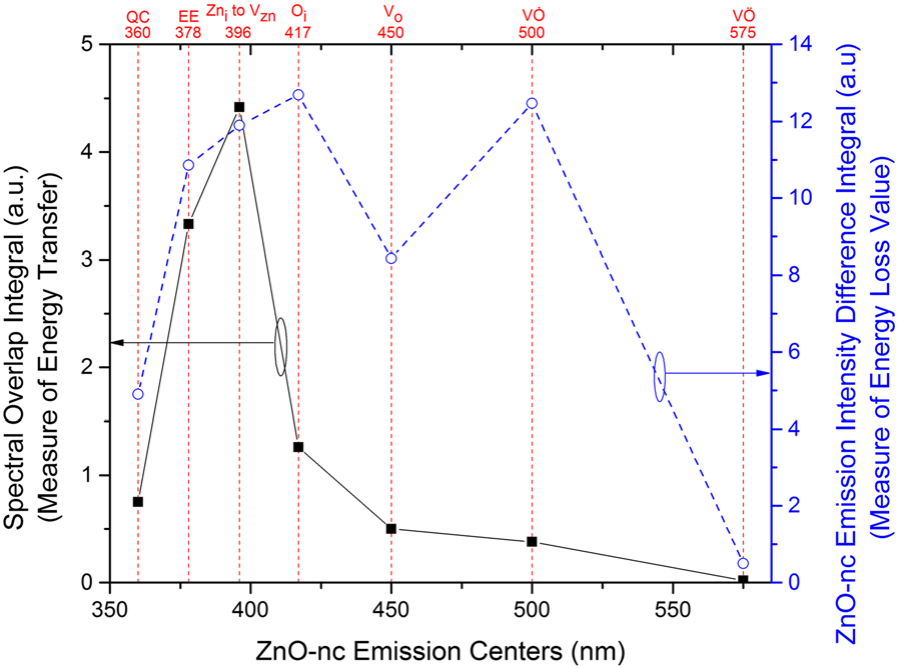
A comparison of the spectral overlap integral and ZnO-nc emission intensity difference integral. The comparison for each of the seven ZnO-nc emission centres for the samples annealed at 450 °C

Furthermore, in Fig. 4a, b, we clearly observe that the emission intensities from various ZnO-nc emission centres in the Eu^3+^_0.12_:(ZnO-nc:SiO_2_) sample are lower than those in the ZnO-nc:SiO_2_ sample. This intensity difference is due to the energy loss from the various ZnO-nc emission centres because of the incorporation of the Eu^3+^ ions. Figure 6 shows the intensity differences of each of the seven ZnO-nc emission centres between ZnO-nc:SiO_2_ and Eu^3+^_0.12_:(ZnO-nc:SiO_2_) samples annealed at 450 °C. Thus, calculating the integral value of the difference in the emission intensities of each of the seven ZnO-nc emission centres in Fig. 6 gives a measure of energy loss from each of the ZnO-nc de-excitation centres in exciting the Eu^3+^ ions. The seven ZnO-nc emission centres intensity difference integral are also shown in Fig. 7 on the right axis (dotted line).

In Fig. 7, we now have the measure of the energy transfer, which is the spectral overlap integral value, and the measure of energy losses, which is ZnO-nc emission centre’s intensity difference integral value, from each of the seven ZnO-nc emission centres. Here, we observe an identical trend between the spectral overlap integral and ZnO-nc emission intensity difference integral values from the QC, EE and Zn_i_ to V_Zn_ ZnO-nc emission centres which are centred at 360, 378 and 396 nm, respectively. Amongst these, the EE and Zn_i_ to V_Zn_ have the highest spectral overlap integral and ZnO-nc emission intensity difference integral values of energy transfer. This implies that the EE and Zn_i_ to V_Zn_ ZnO-nc emission centres contribute the most to energy transfer from ZnO-nc to the Eu^3+^ ions. In contrast, we observe that the ZnO-nc emission intensity difference integral values from ZnO-nc emission centres like O_i_, V_o_ and VȮ centred at 417, 450 and 500 nm, respectively, are higher than the spectral overlap integral values. We propose that this is due to defect centres induced by the incorporation of the Eu^3+^ ions in the Eu^3+^_0.12_:(ZnO-nc:SiO_2_) sample, providing non-radiative de-excitation paths for O_i_, V_o_ and VȮ emissions. This means that only some portion of the O_i_, V_o_ and VȮ ZnO-nc emissions transfer the energy to the Eu^3+^ ions. A large portion of the energy from the O_i_, V_o_ and VȮ ZnO-nc emissions decays non-radiatively through the Eu^3+^ ion-induced defect centres. In this way, even though the spectral overlap integral values of O_i_, V_o_ and VȮ ZnO-nc emissions are low, their ZnO-nc emission intensity difference integral values can be relatively high since these de-excitations occur via the Eu^3+^ ion induced defect states.

## Conclusions

In conclusion, we have convincingly shown that efficient energy transfer takes place from excited ZnO-nc to Eu^3+^ ions embedded in a SiO_2_ matrix. This energy transfer gives strong red emission at 614 nm from the Eu^3+^ ions due to the ^5^D_0_ → ^7^F_2_ transition. This was observed using the PL emission spectra of the abovementioned samples which were optically excited using a continuous excitation at 325 nm. The energy transfer from the ZnO-nc to the Eu^3+^ ion was further confirmed by studying the photoluminescence excitation spectra of the samples. The dependence of the Eu^3+^ red emission on the annealing temperature and also on the Eu^3+^ concentration in the sample was studied and optimised for an annealing temperature of 450 °C and for a Eu^3+^ concentration of 12 mol%. We present a detailed study of the energy transfer by identifying the contribution of the seven different emission centres of ZnO-nc in exciting the Eu^3+^ ions. We clearly show that the EE and Zn_i_ to V_Zn_ ZnO-nc emission centres have the highest contribution to the energy transfer from ZnO-nc to the Eu^3+^ ions. While the O_i_, V_o_ and VȮ ZnO-nc emission centres have low energy transfer contributions but high energy loss due to the presence of Eu^3+^ ions induced defect centres in the Eu^3+^_0.12_:(ZnO-nc:SiO_2_) sample. By understanding the mechanism of energy transfer from ZnO-nc to Eu^3+^ ions and optimising the fabrication parameters in producing the highest red emission from Eu^3+^ ions, we can make future proficient red luminescent solid state devices based on a low-cost technique. Extending these very important analyses to systems with other rare earth elements will also help in making efficient light sources for various applications in photonics.
